# Supplementary material for: Major Stressful Life Events and the Risk of Pancreatic, Head and Neck Cancers: A Case–Control Study
Source: Cancers (Basel). 2024 Jan 20;16(2):451. doi: 10.3390/cancers16020451 (PMC10814511; doi:10.3390/cancers16020451)
Supplement: Supplementary file 1 [file cancers-16-00451-s001.zip › cancers-2784823-supplementary.pdf]

MAJOR STRESSFUL EVENTS (SE) AND RISK OF DEVELOPING LUNG, HEAD/NECK AND PANCREATIC CANCER

(B-14-104/HSC-14-0568)

STUDY QUESTIONNAIRE (CASES)

Step 1: Patient Information

Serial Number	
Patient Identifier (MR #)	
Date of Birth (MM/DD/YYY)	
Gender (circle one)	Male Female
Ethnicity (circle one)	White not Hispanic African-American Hispanic<br>Asian Other
Height (inches)	
Weight (pounds)	
BMI	
Maximum education	Some school High school Some college<br>College degree Masters PhD
Marital status	Married Single Separated<br>Divorced Widowed
Family history of lung, head/neck or pancreatic cancer	Yes No
If yes, please list family member/s with cancer	

UTHealth The University of Texas Health Science Center at Houston
IRB NUMBER: HSC-MS-14-0568
IRB APPROVAL DATE: 09/28/2017

Step 2: General Health Information

Do you have any of the following diseases?

Diabetes mellitus	Yes No
High blood pressure<br>(hypertension)	Yes No
Chronic obstructive pulmonary disease (COPD)	Yes No
Inflammatory arthritis/SLE<br>(rheumatoid arthritis)	Yes No
Cardiovascular disease<br>(heart disease)	Yes No
Major Depression	Yes No

Step 3: Medications (to be completed by staff member)

Is the patient taking any of the following medications?

Beta-blockers	Yes No
Metformin	Yes No
NSAIDS	Yes No
ACE inhibitors	Yes No
Statins	Yes No
Antidepressants<br>(SSRI, SNRI, TCA, MAOI, etc.)	Yes No
Anxiolytics<br>(benzodiazepines, antipsychotics, barbiturates, hydroxyzine, etc.)	Yes No

UTHealth The University of Texas Health Science Center at Houston
IRB NUMBER: HSC-MS-14-0568
IRB APPROVAL DATE: 09/28/2017

Step 4: Smoking History

Have you ever been a smoker?	Yes No
How old were you when you started smoking?	
How old were you when you quit smoking?<br>(Leave blank if current smoker)	
For how many years in total did you smoker?	10-19 years<br>20-29 years<br>30-39 years<br>40-49 years<br>≥ 50 years
How many cigarettes did you typically smoke each day during this time period? (circle one)	<1 pack/day<br>≥1 to < 2 packs/day<br>≥ 2 to < 3 packs/ day<br>≥ 3 packs/day

To be completed by staff:

Smoking exposure (years)	
Smoking exposure (years * cigarettes/day)	

UTHealth The University of Texas Health Science Center at Houston
IRB NUMBER: HSC-MS-14-0568
IRB APPROVAL DATE: 09/28/2017

Step 5: Life Events (Modified from Holmes and Rahe stress scale)

Have any of the following events ever happened in your life prior to lung cancer diagnosis? If Yes, please chose:

A) In past 5 years (B) in past 10 years or (C) Ever?

Death of spouse	Yes No	A) B) C)
Death of child or immediate family member<br><br>Please circle child or family member	Yes No	A) B) C)
Serious life-threatening personal illness other than lung, head/neck or pancreatic cancer	Yes No	A) B) C)
Serious life-threatening illness of an immediate family member	Yes No	A) B) C)
Divorce or separation	Yes No	A) B) C)
Loss of job	Yes No	A) B) C)
Being caregiver to a seriously ill family member	Yes No	A) B) C)
Significant financial difficulties	Yes No	A) B) C)
Move to different city/state/country	Yes No	A) B) C)
Significant stress at Job	Yes No	A) B) C)
Detention/Incarceration	Yes No	A) B) C)
Retirement	Yes No	A) B) C)

To be completed by staff:

Life events score	

UTHealth The University of Texas Health Science Center at Houston
IRB NUMBER: HSC-MS-14-0568
IRB APPROVAL DATE: 09/28/2017

Step 6: UNC-Duke Social Support

Please circle one of the choices for each question.

I have people who care what happens to me.	As much as I would like<br>Almost as much as I would like<br>Some, but would like more<br>Less than I would like<br>Much less than I would like
I get love and affection.	As much as I would like<br>Almost as much as I would like<br>Some, but would like more<br>Less than I would like<br>Much less than I would like
I get chances to talk to someone about problems at work or with my housework.	As much as I would like<br>Almost as much as I would like<br>Some, but would like more<br>Less than I would like<br>Much less than I would like
I get chances to talk to someone I trust about my personal or family problems.	As much as I would like<br>Almost as much as I would like<br>Some, but would like more<br>Less than I would like<br>Much less than I would like

UTHealth The University of Texas Health Science Center at Houston
IRB NUMBER: HSC-MS-14-0568
IRB APPROVAL DATE: 09/28/2017

I get chances to talk about money matters.	As much as I would like<br>Almost as much as I would like<br>Some, but would like more<br>Less than I would like<br>Much less than I would like
I get invitations to go out and do things with other people.	As much as I would like<br>Almost as much as I would like<br>Some, but would like more<br>Less than I would like<br>Much less than I would like
I get useful advice about important things in life.	As much as I would like<br>Almost as much as I would like<br>Some, but would like more<br>Less than I would like<br>Much less than I would like
I get help when I am sick in bed.	As much as I would like<br>Almost as much as I would like<br>Some, but would like more<br>Less than I would like<br>Much less than I would like

To be completed by staff:

Social support score	

UTHealth The University of Texas Health Science Center at Houston
IRB NUMBER: HSC-MS-14-0568
IRB APPROVAL DATE: 09/28/2017

Step 7: Belief System

Do you practice a religion or belief system in your daily life?	Very strongly<br>Strongly<br>Somewhat<br>Occasionally<br>Never
Do you believe in an afterlife?	Very strongly<br>Strongly<br>Somewhat<br>Occasionally<br>Never
Does your practice of a religion or belief system give you hope?	Very strongly<br>Strongly<br>Somewhat<br>Occasionally<br>Never
Do you regularly (at least once a month) visit a place or worship?	Yes<br>No
Do you have associates or friends from your place of worship?	Yes<br>No

To be completed by staff:

Belief system score	

Cancer History (Cases Only):

1. Date of diagnosis: ____ / ____ / ____

2. Histology: (1) Adenocarcinoma
   (2) Squamous cell carcinoma
   (3) Large cell carcinoma
   (4) Adenocarcinoma in situe
   (5) Poorly differentiated carcinoma
   (6) Small cell carcinoma
   (7) Other

3. Stage at diagnosis:
   T= ____________
   N= ____________
   M= ____________

4. (1) Stage I (2) Stage II (3) Stage III (4) Stage IV

5. Molecular studies:
   (1) K-ras 1) Yes 2) No
   (2) HPV p(16) 1) Yes 2) No
   (3) Other mutations ___________________________

6. Treatment: ____

7. Surgery:
   a) _______________________
   b) ________________________
   c) ________________________

UTHealth The University of Texas Health Science Center at Houston
IRB NUMBER: HSC-MS-14-0568
IRB APPROVAL DATE: 09/28/2017

1. Chemotherapy:

(1) Adjuvant

(2) Concurrent with radiation

(3) Neo-adjuvant

(4) Palliative

Chemotherapy regimen:

a) ___________________________________

Number of chemotherapy cycles __________

b) ___________________________________

Number of chemotherapy cycles __________

c) ___________________________________

Number of chemotherapy cycles __________

d) ___________________________________

Number of chemotherapy cycles __________

8. Radiation:

(a) Curative

Dose _____________

(b) Palliative

Dose _____________

Patient's Physiological function at diagnosis:

A) Weight loss prior to diagnosis

(1) _________________ lbs

(b) Percentage weight loss as compared to diagnosis

(1) No weight loss (2) <5% (3) >5%

UTHealth The University of Texas Health Science Center at Houston
IRB NUMBER: HSC-MS-14-0568
IRB APPROVAL DATE: 09/28/2017

B) CBC

Hb ________________mg/dL

WBC ________________k/cmm

Absolute neutrophil count ________________k/cmm

Absolute lymphocyte count ________________k/cmm

Platelet ________________k/cmm

C) Serum Albumin ________________g/dL D

D) Serum creatinine ________________mg/dL

E) CA 19-9 ________________U/mL
